# Novel oleogel formulation based on amaranth oil: Physicochemical characterization

**DOI:** 10.1002/fsn3.1018

**Published:** 2019-05-17

**Authors:** Elahe Kamali, Mohammad Ali Sahari, Mohsen Barzegar, Hassan Ahmadi Gavlighi

**Affiliations:** ^1^ Department of Food Science and Technology Tarbiat Modares University Tehran Iran

**Keywords:** amaranth oil, oleogel, physicochemical, X‐ray diffraction

## Abstract

This study aims to investigate the characteristics of oleogel (OG) produced from amaranth oil at four concentrations of 7%, 9%, 10%, and 12% of the monoglyceride (MG). The physicochemical and structural aspects were performed by using pulsed nuclear magnetic resonance, differential scanning calorimetry, X‐ray diffraction, and gas chromatography. The results show that oleogels (OGs) had higher oxidative stability during storage at ambient temperature in comparison with amaranth oil. Moreover, polarized optical microscopy revealed that an increase in percentage of the oleogelator leads to the formation of needle‐shaped crystals followed by oil entrapment. Also, MG improves the solid content of amaranth oil from 0.5% to 11% and creates a solid structure in spite of the low solid fat as compared to cocoa butter (CB) (82%), as control sample. Crystals similar to CB were also observed when evaluating the crystalline structure of the OG. The fatty acid ratio and the essential linoleic fatty acid were preserved in the OG by only 2%–6% reduction.

## 1 INTRODUCTION

Vegetable oil fats have an important role in foods' nutritional quality, shelf‐life, flavor, and texture (Ogutcu, Arifoglu, & Yilmaz, 2015). However, it should be noted that vegetable oils are more susceptible to oxidation, which might result in rancidity when stored. In addition, they do not perform well in food processing due to their low viscosity (Kim, Lim, Lee, Hwang, & Lee, 2017). Overall, medical guidelines mostly recommend people throughout the world to reduce consuming saturated fats (<10% calories per day). Therefore, it has recently been suggested that oleogels (OGs) be replaced by solid‐like materials which are made of lipids rich in unsaturated fatty acids (O'Sullivan, Barbut, & Marangoni, 2016). OGs are in fact generated from the entrapment of liquid oils in a three‐dimensional network without any alterations in the chemical features of the oil. Although oleogelation is a novel topic, oils as the materials that can be structured into self‐standing solids have been highly attractive during the last decade and have been suggested as hydrogenated or saturated fat replacers, oil binders, inhibitors of oil migration, and oxidation protection systems (Manzocco et al., 2017).

Oleogels are classified based on the methods of processing, molecular nature, and number or chemical type of the organogelators used (Doan et al., 2016). A compound should have certain physicochemical properties to be considered as an oleogelator, including the affinity for oil known as lipophilicity, self‐assembling properties, surface activity, capacity for higher structural arrangement, and exhibiting thermoreversible properties (Patel, Schatteman, Vos, Lesaffer, & Dewettinck, 2013). Emulsifiers could be the most successful group of structuring agents in future and are able to self‐assemble in different environments due to their “schizophrenic” character (Valoppi, Calligaris, Barba, & Nicoli, 2015). In addition, OGs can be categorized based on their oil‐gelling molecules' size. The most common converters are lyotropic liquid crystallines made of monoglycerides (MGs), which are low‐molecular‐weight gelators (Cegla Nemirovsky, Aserin, & Garti, 2015).

In general, MG molecules arrange as inverse lamellas in oil with hydrophilic groups of the glycerol head toward the insides of the MG bilayers forming a two‐dimensional close pack known as 2D hexagonal lattice (Lopez‐Martinez, Charó‐Alonso, Marangoni, & Toro‐Vazquez, 2015) and the hydrophobic chains of hydrocarbons connected with the oil (Ojijo, Neeman, Eger, & Shimoni, 2004). A high amount of a third lipophilic component, such as oil, is entrapped by the shell made of the mentioned MG lamellar bilayers (Valoppi et al., 2015). This arrangement of the MGs creates organogels in vegetable oils, which are stabilized by the hydrogen bonds (between the secondary and primary ‐OH groups all over the bilayers): This results in crystallization of the aliphatic tails in the MGs expanding more stable polymorphs (Lopez‐Martinez et al., 2015). Temperature fall to below 75°C may result in hexagonally packed α‐forms of MGs during the crystallization process. When the temperature drops to below ~50 and ~30°C, thermally reversible polymorphic phase transitions occur and the α‐form undergoes changes in two different sub‐α phases. The α phase and these two sub‐α phases are thermodynamically unstable and gently transform into a triclinic packed β phase, with an elevated melting temperature of about 80°C (Wang, 2016). Practically, the final result is a fat‐like gel that can replace the saturated fat reducing the total and trans fat in a food product (Valoppi et al., 2017). For instance, partial replacement of animal fat in frankfurter sausages by OGs prepared with MGs and phytosterols has been performed (Kouzounis & Lazaridou, 2017). Also, olive oil OG can be structured with monopalmitin and monostearin, creating a spreadable food product (Ojijo, Neeman, et al., 2004). Further, Ogutcu and Yilmaz (2014) suggested that OGs of MGs can be applied as spreadable, breakfast margarine‐like products and augment consumption habits for this healthy oil.

Amaranth, also known as Amaranthus, is among the oldest crops in the world (Czaplicki, Ogrodowska, Zadernowski, & Derewiaka, 2012). Compared to cereals like rice, amaranth grains contain about 30% more protein and are comparable to common grains such as wheat germ regarding minerals such as calcium and manganese. Reduction in blood pressure and cholesterol levels in addition to improvement in the antioxidant status and immunity might also be observed following regular amaranth consumption (Inglett, Chen, & Liu, 2015). On the other hand, multiple reported biological effects (Martirosyan, Miroshnichenko, Kulakova, Pogojeva, & Zoloedov, 2007; Sirota, Yelisyeyeva, Khunderyakova, & Makhotina, 2008; Yelisyeyeva et al., 2014) have been suggested as consequences of the activation of aerobic metabolism (Yelisyeyeva et al., 2012). Additionally, amaranth seed oil may have some benefits for those with cardiovascular and hypertension diseases (Inglett et al., 2015). Moreover, the biologically active composition of amaranth is a part of unsaponifiable oil substances (squalene, tocopherols, and tocotrienols) (Czaplicki et al., 2012).

The aim of this study was to investigate the characteristics of OG produced from amaranth oil. To the authors' knowledge, this was the first time that amaranth oil OG was prepared for use in foodstuffs. Also, cocoa butter (CB) was chosen as control sample because the experience of previous studies shows that CB replaced with vegetables oil presents problems of quality and is unsuccessful as a practical product. Therefore, the present study has been planned to mimic CB properties and establishment of a solid structure such as 3‐D networks in liquid oil with desirable nutritional characteristics by only oleogelators and without hydrogenation or raising the trans‐fatty acids.

## 2 MATERIALS AND METHODS

### 2.1 Materials

Amaranth oil was purchased from Behave Company (Rietberg, Germany), and MG was obtained from Minoo Chocolate Factory (Tehran, Iran). Fatty acid composition of amaranth oil and MG, based on gas chromatography analysis, consisted of about 80% unsaturated and 90% saturated glycerides, respectively. Moreover, both oil and MG were maintained at –24°C until further analysis.

### 2.2 Preparation of the oleogels

Oleogel was prepared with amaranth oil (solvent) and a mixture of MGs of palmitic and stearic acid. MG oleogels were prepared by adding oleogelator to amaranth oil at a concentration of 7%, 9%, 10%, and 12% (wt/wt), which were assigned to OG7, OG9, OG10, and OG12, respectively. The mixtures were heated and stirred (Heidolph MR 3001, Germany) to ~65°C to ensure the oleogelators melted completely. MG OGs were cooled and allowed to crystallize at refrigerator temperature for 24 hr and under static condition (Lupi, Gabriele, Cindio, Sánchez, & Gallegos, 2011).

### 2.3 Determination of oxidation

Peroxide value (PV), represented as mEq O_2_/kg, was measured by the AOCS method (2005) Cd 8b‐90. To study the effect of storage, the OGs were kept at 5 and 25°C for 8 weeks and PV was determined periodically (AOCS, 2005).

### 2.4 Crystal morphology

Olympus DP72 digital camera connected to an Olympus BX51 light microscope operated with Nomarski differential interference contrast and polarized light optics was used to observe crystal morphology. One drop of the sample was located in the middle of a glass slide, and a glass cover slip was placed over it. The crystals were photographed at 100 and 10 times magnification at ambient temperature. Images were achieved and processed using the cell sense (application software) and were saved with a 1,360 × 1,024 pixels resolution in jpeg format (Batte, Wright, Rush, Idziak, & Marangoni, 2007).

### 2.5 Pulsed nuclear magnetic resonance (pNMR)

The solid fat content (SFC), which is regarded as a factor for evaluating melting behavior, was assessed by pNMR using a Bruker Minispec mq20 (Bruker, Karlsruhe, Germany) at various temperatures. Following complete melting at 60°C for 5 min and chilling at 0°C for 60 min, the samples were placed in pNMR tubes and submitted to the IUPAC 2.150 serial tempering method. The SFCs were measured at temperature intervals of 5°C in the range of 5–40°C (Zarringhalami, Sahari, Barzegar, & Hamidi‐Esfehani, 2010).

### 2.6 Differential scanning calorimetry

Thermal properties such as crystallization and melting profiles were determined using Netzsch 200F3 differential scanning calorimetry (DSC). Nitrogen was used as the purging gas at flow of 100 ml/min. Ten mg of the sample was first placed in an aluminum pan and then sealed hermetically with a sample press (the reference was an empty pan to acquire the baseline settings). Afterward, it was heated at 130°C for 3 min to warrant homogeneity and eliminate any previously shaped crystals. The sample was then cooled at 10°C/min to –30°C and held for 3 min, followed by heating at 10°C/min to 130°C: This was maintained for 3 min. The peak temperatures, melting point, crystallization onset, and enthalpies were calculated for each peak by the Netzsch software (Sonwai, Podchong, & Rousseau, 2017).

### 2.7 Polymorphism

X'Pert PRO MPD X‐ray diffraction (PANalytical Company, Poland) fitted with Cu‐Kα radiation (*k* = 1.5406 Å, current of 40 mA and voltage of 40 kV) was used to identify the polymorphic transformations of the samples at room temperature. To melt all the existing crystals and nuclei before the measurement, the samples were heated at 70°C for 5 min. Next, they were analyzed at 2*θ* angles of 1–30° with a scan rate of 5°C/min. HighScore Plus software was applied to analyze the X‐ray diffraction patterns. Assignment of polymorphs was based on the following short spacing properties of CB: α form (*d* = 4.15 Å), β′ forms (*d* = 3.8–4.3 Å), and β forms (*d* = 4.5–4.6 Å). Prior to measurements, the samples were kept at 35°C for 24 hr in order to prevent any polymorphism transition during the analysis (Zarringhalami et al., 2010).

### 2.8 Fatty acid composition

One microliter of methyl ester‐converted fatty acid solution (according to the AOCS methods, 2005) was injected into a gas chromatograph (Agilent, 7890A), which was fitted with a flame ionization detector and a capillary column of Hp‐88 (100 m × 0.25 mm i.d. × 0.2 µm film thickness). Helium was applied as the ultrapure carrier gas with a 3 ml/min flow rate and 1/100 split injection ratio. Temperatures of the injector and detector were 250 and 280°C, respectively. The initial column temperature was fixed to 120°C and kept at this level for 1 min, followed by a heating rate of 10°C/min to a temperature of 170°C for 10 min: Finally, it raised again from 170 to 210°C at a rate of 5°C/min which was maintained for 27 min. Comparison of the retention times with the standards (Sigma, USA) was the approach for recognizing the fatty acid methyl esters. The yield of fatty acids was calculated as follows: area of the fatty acid/area of total fatty acids in the sample of oil × 100 (%) (Zoue, Bedikou, Faulet, Gonnety, & Niamke, 2012).

### 2.9 Statistical analysis

The oleogels were prepared in triplicate, and all analytical procedures were also made in triplicate and SPSS 19.0 program (Chicago, IL, USA) was used for statistical analysis. Significant difference among samples was examined from one‐way analysis of variance (ANOVA), followed by Duncan's multiple range test to compare the means that showed significant variation (*p* < 0.05).

## 3 RESULT AND DISCUSSION

### 3.1 Oleogel preparation

In this study, the optimum range of the oleogelator in oil for generating the OGs was obtained by examining the concentrations of 1%–50%. However, a gel is often considered as a semisolid material composed of low concentrations of organogelator (≤15%) (Cerqueira et al., 2017), although numerous studies have used high organogelator amounts (Cerqueira et al., 2017; Lupi et al., 2016; Singh, Pramanik, Ray, & Pal, 2015). In addition, utilizing a wide gelator concentration range provides the possibility to understand the impact of organogelator molecular properties on the OG characteristics (Cerqueira et al., 2017). Afterward, melting point, texture, and PV were used in order to choose optimal concentrations among the 50 samples (results not shown).

It was observed that gelation occurs at the ≥6% w/w concentration of MG, and that lower MG percentages were insufficient for full entrapment of the liquid amaranth oil. At these low concentrations, integrity could not be maintained when the tubes were inverted which is the primary requirement for a gel (Singh et al., 2014). Augmented MG levels resulted in enhanced gel properties, such as formation, consistency, and smoothness. Therefore, the gelled system improved remarkably and no signs of oiling out or crystalline phase sedimentation were observed over 4–8 weeks of storage. Similar behavior was observed for OG of sunflower oil, phytosterols, and MG by increasing the MG proportion (Sintang, Rimaux, De Walle, Dewettinck, & Patel, 2017). Consequently, the four percentages of 7, 9, 10, and 12 were selected as the optimal ones for other characteristics. On the other hand, the texture hardness and melting point, as compared to CB, could be considered limitations at concentrations higher than 12%.

### 3.2 Oxidative stability

The oxidative stability of the OG samples was measured by monitoring PV for 2 months stored at 4 and 25°C, respectively (Table 1). These findings can be of remarkable importance in commercial production of organogels (Yilmaz & Ogutcu, 2014). Among all OG samples, the lowest (0.8 mEq O_2_/kg) and the highest PV (2.1 mEq O_2_/kg) was obtained in the oil stored at 4°C for 0 days and at 25°C for 60 days, respectively. On the other hand, the range of PV values was from 0.8 mEq O_2_/kg for the sample at the beginning of the storage at 4°C to 1.1 mEq O_2_/kg at the end of it: There was no significant change in PV during the storage time at refrigeration temperature (4°C). However, the samples stored at room temperature had greater PV as compared to the refrigerated samples. Yilmaz and Ogutcu (2014) reported the same results for hazelnut oil and beeswax OGs. Also, PV values of samples at room temperature were statistically different, especially between oil and OGs: Specifically, OG10 and OG12 had lowest values (1.6 mEq O_2_/kg). On the other hand, a gradual elevation in the PVs was observed at both temperatures throughout the storage time. Interestingly, an increase in the PV of fresh oil was significantly faster during storage as compared to the OGs: These findings are in line with Lim, Hwang, and Lee (2017). Moreover, the PV decreased by increasing the concentration of OGs. This suggests that delayed oil oxidation during storage might result from limited oil mobility due to organogelation (Lim et al., 2017). The PVs of all OGs were shown to be below 2.1 mEq O_2_/kg, which is demonstrative of good oxidative stability in the samples. Since no antioxidant was added to the samples, this stability could be attributed to the natural antioxidant compounds (tocopherols, tocotrienols, and sterols) of the oil. Therefore, oleogelation has not led to degradation of these components, which can be counted as another advantage of this technology (Ogutcu & Yilmaz, 2014).

**Table 1:** Peroxide values of the amaranth oil and oleogels stored for 8 weeks at 4 and 25°C

Peroxide value	4°C							25°C						
Day	0	1	3	7	14	28	56	0	1	3	7	14	28	56
Amaranth oil	0.80 ± 0.02^a^	0.80 ± 0.00^a^	0.82 ± 0.04^a^	0.81 ± 0.01^a^	0.95 ± 0.03^a^	0.92 ± 0.02^a^	1.01 ± 0.06^a^	0.92 ± 0.03^a^	0.92 ± 0.01^a^	1.01 ± 0.01^a^	1.20 ± 0.03^b^	1.65 ± 0.01^e^	1.86 ± 0.08^c^	2.10 ± 0.11^c^
OG7	0.81 ± 0.01^a^	0.81 ± 0.02^a^	0.93 ± 0.01^a^	0.94 ± 0.03^a^	0.91 ± 0.00^a^	1.11 ± 0.02^a^	1.10 ± 0.01^a^	0.91 ± 0.03^a^	0.92 ± 0.01^a^	1.00 ± 0.04^a^	1.11 ± 0.06^ab^	1.43 ± 0.02^d^	1.61 ± 0.04^b^	1.91 ± 0.09^b^
OG9	0.91 ± 0.01^a^	0.92 ± 0.05^a^	0.91 ± 0.02^a^	0.94 ± 0.03^a^	1.01 ± 0.01^a^	1.02 ± 0.03^a^	1.11 ± 0.00^a^	0.90 ± 0.07^a^	0.92 ± 0.01^a^	1.02 ± 0.00^a^	1.14 ± 0.02^a^	1.36 ± 0.05^c^	1.52 ± 0.02^b^	1.82 ± 0.01^b^
OG10	0.90 ± 0.03^a^	0.91 ± 0.02^a^	0.92 ± 0.03^a^	1.00 ± 0.04^a^	1.00 ± 0.02^a^	1.03 ± 0.05^a^	1.02 ± 0.04^a^	0.92 ± 0.07^a^	0.93 ± 0.03^a^	1.03 ± 0.07^a^	1.15 ± 0.00^ab^	1.10 ± 0.01^a^	1.31 ± 0.08^a^	1.62 ± 0.00^a^
OG12	0.92 ± 0.01^a^	0.91 ± 0.01^a^	0.90 ± 0.02^a^	1.01 ± 0.06^a^	1.00 ± 0.01^a^	1.00 ± 0.03^a^	1.03 ± 0.04^a^	0.93 ± 0.02^a^	0.91 ± 0.05^a^	1.01 ± 0.03^a^	1.12 ± 0.04^ab^	1.21 ± 0.04^b^	1.31 ± 0.03^a^	1.63 ± 0.01^a^

Note

Each value represents the mean ± standard deviation of three samples. Means (in the same column) with different letters are significantly different (*p* < 0.05)

### 3.3 Polarized light microscopy

**Figure 1:**
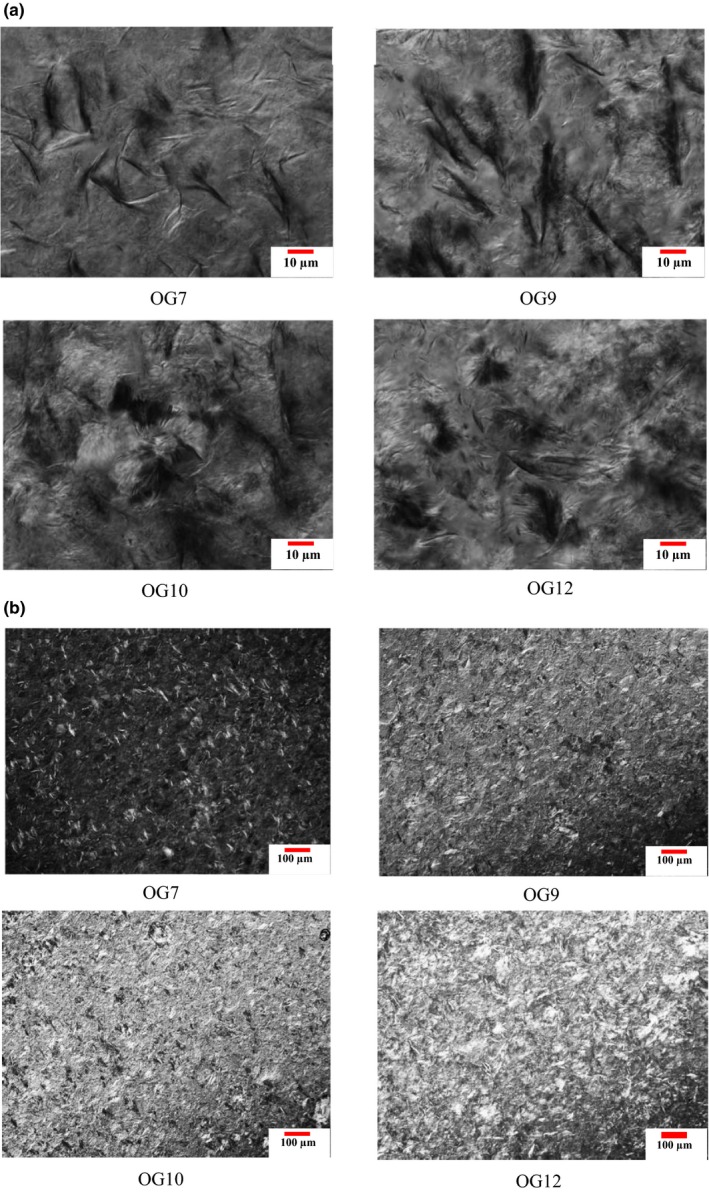
The polarized light microscopy images of the oleogel (OG) samples, (a) OG7, OG9, OG10, and OG12 at ×10 magnification and (b) the same samples at ×100

Cooling of the mixture gave rise to the crystal phase and caused a polydomain crystal network to form. Organogel formation process occurs in three steps: crystal nucleation, crystalline branching, and crystal growth (Cerqueira et al., 2017). The microphotographs of polarized light microscopy (PLM) acquired for OG7, OG9, OG10, and OG12 are shown in Figure 1. Needle‐like crystals were revealed in PLM microphotographs similar to the results observed by Kesselman and Shimoni (2007) in commercial MG crystallized in olive oil at 25°C by Ojijo, Kesselman, et al. (2004); Co, and Marangoni, (2012); Rocha‐Amador et al. (2014) and Da Pieve, Calligaris, Eco, Nicoli, and Marangoni (2010). Also, Sintang et al. (2017) mentioned that MGs cluster as crystal aggregates along with one‐dimensional needle crystals and spherulite crystallites in OGs can be obtained by using different sources such as structurants at 10% concentration. The spherulites result from aggregation of the needle‐like crystals, grown from a nucleation center with several branches that form large crystal structures (Sintang et al., 2017). Palla, Giacomozzi, Genovese, and Carrin (2017) obtained micrographs, which show irregular elongated, fibrillary, and needle‐like dispersion of Myverol (commercial mixture of saturated MG) aggregates. Besides, light microscopy demonstrated the formation of network‐like structures through entanglement of crystals. Maintenance and diffusion restriction of sunflower oil into the outer part of the structure was shaped by dense crystals along with crystal entanglement (Sintang et al., 2017).

On the other hand, an increase in gelator concentration promotes reorganization of the crystals leading to different microscopy images according to the concentration of gelator used; similar results were observed by Cerqueira et al. (2017) in their study on the effect of oil on organogelator structures. Generally, most organogelators organize mesoscale asymmetric structures and do not grow similarly across the three dimensions (Palla et al., 2017). With an increase in gelator concentration, gelation (based on a continuous network, achieved by cross‐linking of the crystals and trapping of the oil) continues until organization of a 3‐D structure immobilizing the oil phase (Cerqueira et al., 2017). Notably, the MG oleogel concentration of 7 had a microstructure with the lowest mass of crystals and the structure became more compact by increasing the concentration to 12% regardless of the storage time.

### 3.4 Solid fat content

Solid fat content is of significance in hard fat stocks from both the technological and the nutritional standpoints (Ogutcu & Yilmaz, 2014). Table 2 shows its values for amaranth oil, OGs, and CB. It should be mentioned that SFC was lower than the addition levels of the organogelators in OG samples. Ogutcu and Yilmaz (2014) also claimed that some of the organogelators are liquid at some specified temperatures. As expected, amaranth oil has the biggest difference (statistically) with CB and OG12 has the least. In fact, amaranth oil remained liquid at temperatures of 0–40°C with SFC of about zero: Si, Cheong, Huang, Wang, and Zhang (2016) reported the same results for soybean oil. Nonetheless, this percentage is enhanced and a three‐dimensional structure is formed in the oil by adding some MGs with saturated fatty acids to the oil: The final result is generation of solid fats. Lack of trans fat production in this process can be regarded as one of the most remarkable advantages of using oleogelators. Furthermore, Niiya, Imamura, Okada, and Matsumoto (1973) stated that adding saturated MGs to palm kernel oil raises melting point, induces crystallization, and improves solid fat content. However, low temperatures cause CB to be more reach in solid fat. On the other hand, CB has a higher solid fat content at 0–35°C. At 5 and 10°C, SFC for CB was ~82.1% and 80.5%, respectively. This is mainly due to the presence of higher saturated fatty acid content in CB. While CB contained 70.5% SFC at 20°C, the organogels had 5.5%–8.8% SFC. OGs are capable of entrapping oil with lower solid content, leading to gel‐like property at low temperatures (Si et al., 2016). It has been proved that organogelation does not increase oil SFC significantly, evidencing that no chemical change takes place in during fatty acid saturation or isometry: This could be considered the main advantage of organogels (Yilmaz & Ogutcu, 2014). Additionally, a rapid decline in solid fat content is observed at 20–35°C, which is indicative of solid structure maintenance at lower temperatures and melting occurrence by temperature elevation responsible for the favorable feeling in mouth induced by these specimens. The amount of solid fat depends on the fatty acids and temperature: This percent is expected to decrease by augmentation in the temperature. The SFC of all samples diminished to <0.5% at 40°C. Lack of waxiness and complete melting during consumption were warranted by low SFC at body temperature: Si et al. (2016) showed the same behavior in soybean oil OG.

**Table 2:** Solid fat content of the amaranth oil, cocoa butter (CB), and oleogel samples between 0 and 40°C

Temperature (°C)	Amaranth oil	CB	Monoglyceride	OG7	OG9	OG10	OG12
0	0.50 ± 0.01^e^	82.15 ± 0.52^a^	93.03 ± 2.25^a^	6.82 ± 0.43^d^	8.50 ± 0.49^c^	8.90 ± 0.33^c^	11.12 ± 0.81^b^
5	0.50 ± 0.02^e^	80.59 ± 1.26^a^	91.14 ± 2.06^a^	6.41 ± 0.09^d^	7.81 ± 0.52^c^	8.10 ± 0.37^c^	10.43 ± 0.11^b^
10	0.41 ± 0.01^e^	75.75 ± 0.91^a^	85.79 ± 2.10^a^	6.10 ± 0.85^d^	7.51 ± 0.91^c^	7.52 ± 0.42^c^	9.50 ± 0.16^b^
20	0.32 ± 0.00^e^	70.54 ± 2.50^a^	80.75 ± 1.50^a^	5.52 ± 0.34^d^	6.82 ± 0.44^c^	6.93 ± 0.62^c^	8.81 ± 0.98^b^
30	0.11 ± 0.01^e^	25.63 ± 0.67^a^	47.38 ± 1.33^a^	3.11 ± 0.22^d^	4.23 ± 0.13^c^	4.51 ± 0.00^c^	5.62 ± 0.39^b^
35	0.00 ± 0.00^e^	10.31 ± 0.51^a^	26.25 ± 1.16^a^	1.91 ± 0.20^d^	2.62 ± 0.09^c^	2.91 ± 0.10^c^	3.80 ± 0.14^b^
40	0.00 ± 0.00^a^	0.00 ± 0.00^a^	6.04 ± 0.55^a^	0.00 ± 0.00^a^	0.11 ± 0.00^ab^	0.10 ± 0.00^ab^	0.31 ± 0.02^b^

Note

Each value represents the mean ± standard deviation of three samples. Means (in the same column) with different letters are significantly different (*p* < 0.05)

### 3.5 Thermal properties

**Figure 2:**
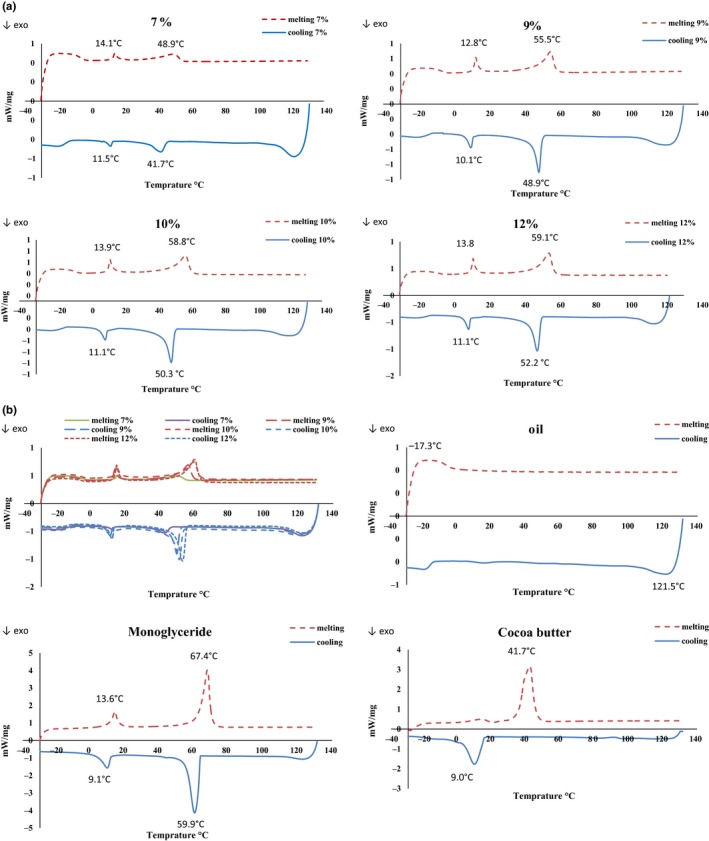
Differential scanning of colorimeter's graphs from (a) comparison of OG7, OG9, OG10, and OG12, (b) amaranth oil, monoglyceride (MG), and cocoa butter (CB)

The graphs obtained from the thermal behavior of comparing OGs, oil, MG, and CB with DSC are shown in Figure 2 for temperatures ranging from −30 to 130°C. Monoglyceride OGs are presented as two peaks at all concentrations. Similar results were obtained in a study conducted by Perez‐Monterroza, Ciro‐Velasquez, and Tobon (2016) that showed that 10% OGs of commercial MGs had two crystallization peaks related to saturated and unsaturated components of MG. These observations were in accordance with the findings of Si et al. (2016). Furthermore, Basso et al. (2010) mentioned that these crystallization peaks may result from the β and α polymorphs, which is also comparable to the results found by Saberi, Lai, and Toro‐Vázquez (2011) in a study on palm oil crystallization with monoacylglycerols and tripalmitin. It is indicated in their study that adding monoacylglycerols increases the number of crystallization seeds, decreases size of crystals, and favors the formation of the β crystals resulting in the accelerated crystallization of palm oil. On the other hand, temperatures of the observed melting peaks elevate with an increase in MG concentrations and reach 59.1°C from 48.9°C by changing the OG percent from 7% to 12%. Si et al. also reported that melting temperature of MG oleogel 3 was about 38.27°C; however, melting temperature of ~46.24°C was observed for MG oleogel 6%. This is in agreement with the previously reported findings of Blake, Co, and Marangoni (2014) who found out that higher ratios of oleogelators in OGs are accompanied by higher melting points. Generally, peak temperatures were shown to have concentration‐dependent decrease versus decrease in MG proportions: This was as expected (Sintang et al., 2017). This finding can find technological application: A suitable level of organogelator can be selected for addition based on thermal properties and other aspects (Yilmaz & Ogutcu, 2014). In addition, a study performed by Perez‐Monterroza et al. (2016) revealed that MGs concentrations might also influence the onset temperature (*T*_onset_) and crystallization temperatures (*T*_c_), probably due to the reciprocal interactions and the triacylglycerols from avocado oil. The oil TAGs contain double bonds, the steric effect of which plays a role in preventing the accommodation of MGs chains, lower *T*_c_ and *T*_onset_, and formation of a less compact structure. The latter result is consistent with studies on crystallization of the candelilla wax OGs using safflower oil rich in triolein (Toro‐Vazquez, Alonzo‐Macias, Dibildox‐Alvarado, & Charo‐Alonso, 2009). Moreover, OG samples demonstrated lower peak temperature as compared to pure MG samples: Maximum peak temperature in these cases was 67.4 and 59.1°C in MG and OG12, respectively. Kouzounis and Lazaridou (2017) reported that increased solubility of the solid crystals can be attributed to the oil medium. A similar trend has been observed on thermal DSC scans of virgin olive oil OGs with saturated MGs in comparison with the thermogram of the pure gelator (Lupi et al., 2016). Further, the peak temperatures were –17.3 and 41.7°C in oil and CB, respectively: This indicates the improving impact of adding MG to oil for increasing the melting point and getting close to the control sample. In fact, the organogels exhibit melting temperatures and enthalpies alike the CB sample. Hence, they can be applied as good alternatives and which will not cause any problem regarding thermal conditions in food processing. In plastic fats, melting temperature is an essential determining parameter for different applications. Moreover, the temperature of the OG peaks was very close during the melting and cooling processes, which confirms that the OGs are completely thermoreversible. Furthermore, execution of thermal cycling during evaluation yields very similar results (Yilmaz & Ogutcu, 2014).

### 3.6 Determination of polymorphic structure

**Figure 3:**
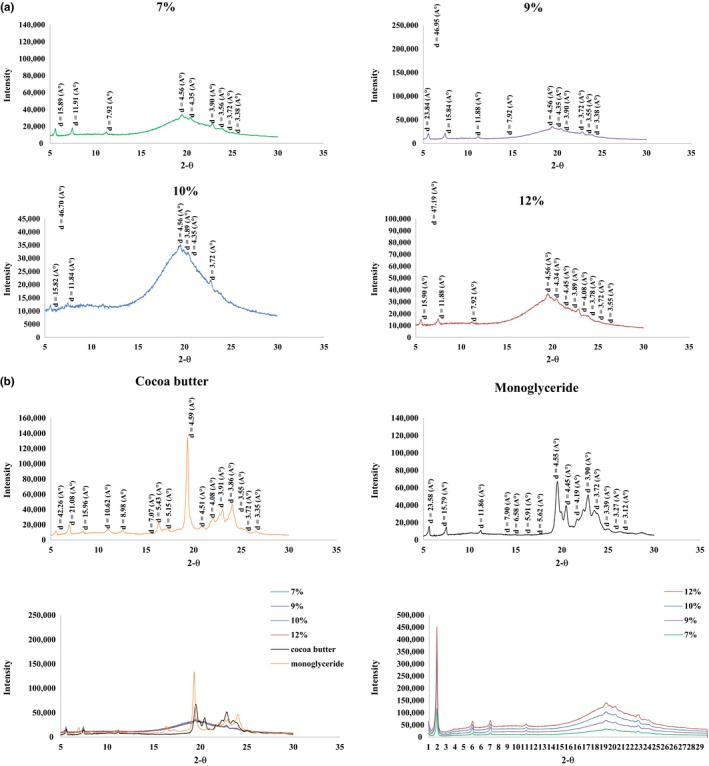
X‐ray diffraction patterns of the (a) comparison of OG7, OG9, OG10, and OG12, (b) cocoa butter (CB), monoglyceride (MG), compare of all samples and compare of oleogels

The type of crystals in short spacing, which is specific to each crystal, has been determined. Figure 3 demonstrates the crystalline structure of OGs, MGs, and CB via a X‐ray diffraction device. Wide‐angle region peaks from 3.12 to 4.59 Å; small‐angle region diffraction peaks at around 5.15–42.26 Å for the MG olegels which were measured: Similar peaks were determined for 5% and 7% MG in cod liver oil organogels (Da Pieve et al., 2010; Da Pieve, Calligaris, Panozzo, Arrighetti, & Nicoli, 2011). Pure oil is free of crystals, and MG has a highly intense peak at transverse distance of 4.55 Å: This is related to the presence of β crystals. Moreover, the unique melting properties and thermal behavior of CB is also due to the presence of a specific type of β crystals at a transverse distance of about 4.6 Å: The α and β′ crystals are formed at 3.8 and 4.2 Å. Therefore, comparing this short spacing with the higher peak at points near 4.6 Å in OGs, the similarity of these gels with CB can be concluded. Perez‐Monterroza et al. (2016) reported that peaks at 3.7, 3.9, 4.1, and 4.2 Å are characteristic of the polymorphic form β′ or that sub‐α were found. Moreover, the signal around 4.55 and 4.6 Å presented the transformation of the β′ polymorph to β polymorphic form. However, it has been established in several researches that the formation of β polymorph is induced by MGs (Basso et al., 2010; Ribeiro et al., 2009; Saberi et al., 2011). The same behavior is observed in commercial MG organogels at concentrations more than 1.5% (Lopez‐Martinez et al., 2014). On the other hand, an increase in enthalpy is also observed (results not shown): This might be due to the presence of β polymorph in the mixtures of glycerol monostearate and miglyol oil. Alternatively, the incorporation of the alkyl chain of GMS into the alkyl chain of miglyol oil can be lead to increased enthalpy. A similar effect is observed for the mixtures of GMS and palm oil, where the GMS50 P sample has higher crystallization enthalpy in comparison with pure oil (Alfutimie, Al‐Janabi, Curtis, & Tiddy, 2016). Further DSC experiments suggest that the β polymorph could crystallize at temperatures of 8–8.5 and 5°C directly from the inverse lamellar α state phase and through a fast polymorphic transition from the sub‐α phase, respectively (Lopez‐Martinez et al., 2014). Smaller d‐spacings diffracted by the β phase or the coagel phase show the triclinic structure of MG molecules, where they are more densely packed as compared to the hexagonally packed α phase (Wang, 2016). Furthermore, these crystals increase in OGs as the result of augmented oleogelator amounts and get closer to CB. Intensity of the peaks increases at higher concentrations of gelators, and alteration of d‐spacing can be explained by structure modification following the elevation in gelator concentration (Cerqueira et al., 2017).

### 3.7 Gas chromatography analysis for fatty acid composition

Percentages of the predominant fatty acids in oil, OG7‐12, and CB measured by gas chromatography are shown in Table 3. As expected, by adding MG, the quantity of unsaturated increases while that of saturated fatty acids decreases. The amount of unsaturated oleic and linoleic fatty acids diminishes to 12% and 6%, respectively, in OG as compared to the oil, while the saturated palmitic and stearic fatty acids reach 14.51% and 8.71%, respectively, in OG12 from 6.55% and 3.37% in oil. MG‐induced changes can lead to a balance among fatty acids and make their proportion closer to that of the CB. MGs mainly consist of palmitic and stearic acid, the longer chains of which show stronger Van der Waals interactions (Blake et al., 2014). Additionally, the saturated fatty acid content of CB is much higher than OG; however, OGs with a stearic acid content of 7.38%–8.71%, as compared to 36.36% in CB, have produced similar solid structures. In addition, the value of essential and unsaturated fatty acids in the OG is close to the original oil and much higher than that of the CB.

**Table 3:** Fatty acid composition of the amaranth oil, cocoa butter (CB), and oleogel samples

Fatty acid (%)	Amaranth oil	CB	Monoglyceride	OG7	OG9	OG10	OG12
16:0	6.55 ± 0.02^e^	26.88 ± 0.21^a^	55.43 ± 1.92^f^	12.44 ± 0.20^d^	13.51 ± 0.53^c^	14.10 ± 0.00^bc^	14.51 ± 0.24^b^
18:0	3.37 ± 0.01^e^	36.36 ± 0.30^a^	34.92 ± 1.54^b^	7.38 ± 0.37^d^	7.89 ± 0.25^cd^	8.45 ± 0.11^cd^	8.71 ± 0.77^c^
18:1	30.21 ± 0.52^b^	32.77 ± 0.76^a^	6.03 ± 0.01^e^	28.58 ± 0.92^bc^	27.95 ± 0.30^c^	27.16 ± 0.81^cd^	26.53 ± 0.49^d^
18:2	51.38 ± 1.75^c^	2.70 ± 0.00^a^	3.77 ± 0.00^a^	50.22 ± 1.41^bc^	48.98 ± 0.91^bc^	48.10 ± 2.18^b^	47.88 ± 1.01^b^

Note

Each value represents the mean ± standard deviation of three samples. Means (in the same column) with different letters are significantly different (*p* < 0.05)

## 4 CONCLUSION

This study aims to investigate the characteristics of OG produced from highly unsaturated amaranth oil and MG as an oleogelator. The mentioned OG is made in order to offer a solid‐like oil without nutritional disadvantages of saturated and trans‐fatty acids. As compared to the oils, the results show a slight increase in the PV of the OG samples and comparatively higher oxidative stability without adding antioxidants. Furthermore, adding oleogelator creates a favorable crystalline network in oil and does not allow it to be released. Moreover, OG formation also leads to a solid structure without augmenting the solid fat content of the oil. On the other hand, OG generation causes the melting point of the oil to improve and similar crystals of CB to form. Evaluation of fatty acids by gas chromatography demonstrates 5%–12% and 2%–6% reduction in oleic and linoleic acids, respectively. As a conclusion, the OG made from amaranth oil and MG can be proposed as a solid‐like oil without trans‐fatty acids.

## ETHICAL STATEMENTS

This study does not involve any human or animal testing.

## CONFLICT OF INTEREST

The authors notify that there are no conflicts of interest.
